# Sources of influence for choosing an operating department practitioner career: Findings from a questionnaire among students in England

**DOI:** 10.1177/17504589241265833

**Published:** 2024-08-15

**Authors:** Lucy Wallis, Maja Palmer, Rachel Locke, James Faulkner, Helen Lowes, Beverley Harden

**Affiliations:** 1 University of Winchester, University of Bristol, Bristol, UK; 2 University of Southampton, Southampton, UK; 3 University of Winchester, Winchester, UK; 4 NHS England, London, UK

**Keywords:** ODP, Operating department practitioner, Career choice, Recruitment, Sources of influence, Students

## Abstract

**Aims:**

The aim of this study was to explore the sources of influence which impact choosing an operating department practitioner career among current operating department practitioner students in England to inform recommendations for maximising recruitment and retention.

**Methods:**

An online questionnaire was disseminated to allied health professional, inclusive of operating department practitioner, students in England in 2021.

**Results:**

One hundred and fifty operating department practitioner students attending undergraduate courses completed the questionnaire. Personal influences, such as role models, were the key sources of influence for choosing an operating department practitioner career. Educational sources were the least influential. Gaining work experience or exposure to the theatre setting was perceived as key to address course attrition. Conducting one’s own research was vital in learning more about the operating department practitioner role and influencing the decision to choose the profession.

**Conclusions:**

There are opportunities to utilise media and educational sources more effectively to influence individuals to choose an operating department practitioner career.

## Introduction

Operating department practitioners (ODPs) are part of the allied health professionals (AHP) group in England and are part of a multidisciplinary team, including nurses, anaesthetists and surgeons (Rodger & Mahoney 2017). They work predominantly in operating theatres but also in other clinical areas including accident and emergency departments (Lowes et al 2020), intensive care and high dependency units (Rodger & Mahoney 2017). Retention of ODPs in England has been recognised as a priority in the recently published NHS Long Term Workforce Plan (NHS England 2023). In addition, the NHS (2022) elective care recovery plan has outlined the vital need to grow the theatre workforce to ensure elective care recovery. Gaining an insight into sources of influences on career choices for the ODP student population helps identify recommendations for maximising recruitment and retention, therefore meeting the vision for ODP workforce development in the NHS. We defined sources of influence as how different means of learning about the profession had impacted on career choice, for example, through social media or a career advisor, as opposed to how participants were learning about the profession. The aim of this study was to explore sources of influence, and the impact of these, to choosing an ODP career for ODP students through a national questionnaire.

## Literature review

There is limited literature exploring career choice made by ODPs and work that has been done has been as single-site studies: Wordsworth (2015) and Ali et al (2022). Wordsworth (2015) administered an online questionnaire to first-year AHP students on several courses, including ODPs, at one Higher Education Institution (HEI) to learn about factors that motivated students to choose their profession. The total number of students was 285, but it is unknown how many of these were ODP students. Similarly, ODP career choice was explored in focus groups with six ODP first-year students as part of a study involving therapeutic and diagnostic radiography and the role of work experience (Ali et al 2022). Both studies mainly focused on how students were learning about the profession as opposed to the impact of these sources. A finding for both studies was that ODP students discovered the profession through a chance experience as a patient (Ali et al 2022, Wordsworth 2015), or a family member working in a related area of work (Wordsworth 2015). Wordsworth (2015) also found that few ODP students had learnt about the career through career advisors. Ali et al (2022) emphasised the challenges of obtaining ODP work experience. Students conducting their own research, including websites and social media, were found to be a key source of influence in learning about the profession (Ali et al 2022). To our knowledge, our study was the first to explore ODP career choice, which was not restricted to a single HEI, and included ODP students from all years of study.

## Methods

An online questionnaire was disseminated nationally to AHP students between February and March 2021. The convenience sample for *this* article comprised students, in all years of study and including apprenticeships, on undergraduate ODP courses in England. This is approximately 27 HEIs (HEE 2022). Dissemination of the questionnaire to universities in England was through Health Education England (HEE) Education Leads for the ODP professional body. In addition, promotion of the questionnaire took place through the HEE website, internal HEE newsletters and social media. The questionnaire was designed and hosted using JISC (Bristol, UK) software and accessed through a link.

Ethical approval was obtained from the University of Winchester’s Research and Knowledge Exchange Ethics Committee (Reference: HWB_REC_21_03). The questionnaire was online and anonymous. Participant information explaining the study and a consent page, comprising a confirmation box, were included at the start. The questionnaire included 26 questions and took approximately 15 minutes to complete.

In terms of validity of the questionnaire, a scoping review (Wallis et al 2023) and focus groups were conducted to inform the design. In addition, the questionnaire was piloted among 50 individuals: undergraduate students at the University of [name redacted for anonymity] Winchester, members of an AHP student leadership programme and AHP professional bodies’ Education Leads. Clarification as to which professions comprise AHPs was added to the questionnaire following feedback.

The questionnaire had demographic questions and three main sections: motivations, sources of influence and barriers to choosing an AHP career. Sources of influence will be the focus of this article owing to this area of our research being most applicable to addressing ODP recruitment and retention. Participants were asked to respond using a 5-point Likert-type scale comprising strongly disagree (1), disagree (2), neutral (3), agree (4) and strongly agree (5). Participants could provide a ‘not-applicable answer’ and were asked to select this option if they had not heard about the ODP profession through this source; the focus was to understand the impact of sources through which individuals were learning about the ODP role. For each section, there was the opportunity to provide further comment through free-text boxes. There were three open questions; however, for the scope of this article, only one question is relevant and therefore included. Thematic analysis utilising Braun and Clarke’s (2019) approach was used to analyse the open question.

### Statistical analysis

The sources of influence were categorised as personal, professional, educational and media/marketing. Variable selection was guided by background knowledge (Heinze et al 2018) gained from experts and the literature, including a scoping review (Wallis et al 2023). An influence was counted if the participant had agreed or strongly agreed to the statement. To carry out the test, the Likert-type scale was dichotomised to 1 = strongly agreed and agreed, 2 = strongly disagreed, disagreed and neutral (Jeong & Lee 2016). The ‘not applicable’ option was set as missing data due to our focus of exploring the influence of sources. How each category was defined can be seen in Table 3. Individuals were excluded only if they had missing gender data or they were not on the undergraduate/apprentice pathway. A total of five respondents were excluded, giving a total analytical sample of 150. Descriptive statistical analysis was carried out using Pearson’s chi-square, which tested the differences between influential factors and gender, and between influential factors and age (under 21 years old vs 21 years old and over). Statistical significance was established at p ⩽ 0.05. The analysis was conducted using SPSS 27.0 statistical package.

## Results

Of the 150 ODP students in England who completed the questionnaire, 47% were in their first year, 26% in their second and 16% in their final year of study. For the 17 participants (11%) on the apprenticeship route, nine were in their first year and eight were in the second year. Demographics of the sample are shown in Table 1. There were 99 participants who answered the question: ‘From what you know now as a student on your course, what advice would you give someone interested in this profession?’. Quotes from answers to this open question are included throughout the findings.

**Table 1:** Participant demographics

Demographic characteristic	Classification	n (%)
Participants (n)		150
Ethnicity	White	118 (78.7%)
	Ethnic minority	32 (21.3%)
Gender	Male	29 (19.3%)
	Female	121 (80.7%)
Age	⩾21 years	128 (85.3%)
	<21 years	22 (14.7%)

Data presented as total number (n) and percentage (%).

Table 2 identifies that 72.7% of participants agreed/strongly agreed that they had been influenced by at least one source (a personal, educational, professional or media source). Personal influence had the lowest number of participants reporting *no* influence and a relatively large spread across the number of personal exposures. This was the only type where participants reported five different types of influential factors. Table 3 explores the impact of individual sources of influence. The results show the importance of personal influences, particularly professional role models (influential for 73.2% of the sample). The results for participants aged under 21 or 21 and over and by gender were also considered, but no statistically significant findings were found. For the open question, speaking to those who knew the role was perceived to be important to understand the profession and gain knowledge about the course:

**Table 2:** Number (and %) of participants agreed/strongly agreed to multiple personal, educational, professional and media influence to ODP

	0^a^	1	2	3	4	5
Influences						
Personal	27 (18.0)	27 (18.0)	41 (27.3)	30 (20.0)	16 (10.7)	9 (6.0)
Educational	92 (61.3)	37 (24.7)	10 (6.7)	6 (4.0)	–	–
Professional^b^	53 (35.3)	58 (38.7)	28 (18.7)	11 (7.3)	0	0
Media and marketing^b^	41 (27.3)	47 (31.3)	39 (26.0)	12 (8.0)	7 (4.7)	–
Total personal, educational, professional and media	41 (27.3)	60 (40.0)	38 (25.3)	11 (7.3)	0	0
Female (*n* = 121)	35 (28.9)	48 (39.7)	30 (24.8)	8 (6.6)	0	0
Male (*n* = 29)	6 (20.7)	12 (41.4)	8 (27.6)	–	0	0
Participants ⩾21 (*n* = 128)	36 (28.1)	50 (39.1)	33 (25.8)	9 (7.0)	0	0
Participants < 21 (*n* = 22)	5 (22.7)	10 (45.5)	5 (22.7)	–	0	0

– cell count under 5.

Gender and age are by total number of influences. There is no statistically significant difference between total number of influences and age of participants nor between gender.

a Number of sources of influence.

b Only three possible exposures.

**Table 3:** Number (and %) of participants who agreed/strongly agreed personal, educational, professional and media sources had influenced their career path into ODP

		Number (%) of total ODP participants^a^	Number (%) of participants ⩾21	Number (%) of participants < 21 years	Pearson chi-square^b^	Number (%) of female participants	Number (%) of male participants^a^	Pearson chi-square^b^
Influence	Source							
Personal	Role model	90 (73.2)	76 (61.8)	14 (11.4)	0.357	74 (60.2)	16 (13.0)	0.451
	Own/relative experience	73 (63.5)	61 (53.0)	12 (10.4)	0.760	63 (54.8)	10 (8.7)	0.447
	Someone working in profession	66 (60.0)	60 (54.5)	6 (5.5)	0.161	51 (46.8)	15 (13.6)	0.064
	Family member	42 (42.9)	37 (37.8)	5 (5.1)	0.929	31 (31.6)	11 (11.2)	0.140
	Friend	37 (37.8)	33 (33.7)	–	0.444	30 (30.6)	7 (7.2)	0.589
Educational	University (first degree)	32 (35.6)	28 (31.1)	–	0.863	26 (28.9)	6 (6.7)	0.826
	Professional visiting school	19 (22.6)	14 (16.7)	5 (6.1)	0.274	16 (19.0)	–	0.789
	Teacher	17 (20.0)	13 (15.3)	–	0.292	15 (17.6)	–	0.477
	Career advisors	16 (19.3)	12 (14.5)	–	0.423	13 (15.7)	–	0.938
	Future careers programme	13 (16.3)	10 (12.5)	–	0.466	10 (12.5)	–	0.662
Professional	Previous work in healthcare	79 (70.5)	75 (67.0)	–	**0.009**	61 (54.5)	18 (16.1)	**0.035**
	Work shadowing	39 (43.3)	33 (36.7)	6 (6.7)	0.775	29 (32.2)	10 (11.1)	0.357
	Voluntary healthcare work	29 (34.1)	17 (20.0)	12 (14.1)	< **0.001**	24 (28.2)	5 (5.9)	0.788
Media and marketing	Universities	98 (72.1)	81 (59.6)	17 (12.5)	0.323	77 (56.6)	21 (15.4)	0.697
	Social media	38 (33.6)	28 (24.8)	10 (8.8)	**0.032**	35 (31.0)	–	0.052
	National bodies	31 (29.2)	26 (24.5)	5 (4.7)	0.568	25 (23.6)	6 (5.7)	0.934
	Careers fairs	29 (30.2)	22 (22.9)	7 (7.3)	0.081	25 (26.0)	–	0.332
	Television	13 (13.7)	10 (10.5)	–	0.289	11 (11.6)	–	0.530
Own research	Own research	122 (86.5)	103 (73.0)	18 (12.8)	0.231	96 (68.1)	26 (18.4)	0.273

– cell count under 5.

a Total number of participants who had answered the survey questions, excluding participants who selected ‘not applicable’.

b Chi-square tested the difference between participants’ age/gender who agreed/strongly agreed compared to those who strongly disagreed/disagreed/neutral to the statement.

*I would advise them to ask questions about the profession and to ask current students about the course, placements.*

*Speak to people already in the profession, and make sure you know what you will be getting yourself into.*

We found that 61.3% of participants reported not to have been influenced by an educational source (Table 2), which was the highest across the type of sources. In terms of individual educational sources (Table 3), 19.3% had been influenced by a career advisor and 16.3% by a future careers programme. These were not mentioned as influential in answers to the open question.

Overall, 35.3% of participants did not view any professional sources as influential (Table 2). However, Table 3 suggests the importance of previous work in healthcare (70.5%) as a source of influence. In terms of gender, findings showed that 90% of male participants who answered the question had been influenced by previous work in healthcare compared to 66.3% of female participants (p = 0.035). In terms of differences between those aged under 21 or 21 and over who had previously worked in healthcare, Table 3 shows this was statistically significant. Of participants aged 21 and over who answered the question, 74.2% agreed/strongly agreed with the statement compared to 36.4% (cell count under 5) of under 21-year-old participants (p = 0.009). Findings from the open question emphasised the importance of prior work in healthcare especially in gaining theatre exposure:

*Work in operating theatres for a year as a support worker in order to familiarise yourself with the operating department environment! It helps a lot!*

*I feel the path I have taken from being a healthcare assistant within theatres to going onto an apprenticeship programme was the best way (personally). Most students on my course have some form of previous history within healthcare which enables them to adjust to the theatre environment quicker whereas those who do not struggle and are at higher risk of leaving the course.*

Of participants aged under 21 years who answered the question, 70.6% agreed voluntary work had influenced their decision compared to 25% of participants 21 and over (p < 0.001). In terms of work shadowing, this was seen as influential for 43.3% of the sample. The challenges of organising and undertaking work experience were identified in the open question:

*Work experience is hard to get. If you’re old enough, whatever course you’re applying for, go and work in a hospital for 3 months. Sign up as a bank HCA [healthcare assistant] or Theatre Support worker etc. It’s a unique environment, see what it’s really like to be there and all the different roles you can have. Don’t let your first day on placement be the first time you’ve ever set foot in a hospital as anything other than a patient.*

Table 2 shows that 72.6% of participants had been influenced by at least one media and marketing source. In terms of information regarding the ODP degree from media and marketing sources, this was mainly obtained from universities (72.1%) and social media (33.6%) (Table 3). Of the participants who answered the question, 55.6% of those aged under 21 years had been influenced by social media compared to 29.5% of participants 21 and over (p = 0.032). With the exception of conducting own research, information obtained from universities was the most reported source.

In terms of conducting research, this was influential for 86.5% of the sample. ‘Conducting own research’ is included as a separate category (Table 3), as this was considered to involve a predetermined idea of what someone is looking for and not an external source of influence. Furthermore, it is likely to include a number of variables, for example, social media or attending open days. Undertaking thorough research into the role was perceived to be crucial to help gain an understanding of an ODP in the open question:

*Do lots of research before starting the course to know what the role involves and what the job will involve after university.*

*Really look into it first before applying. I think some people assume it will be more hands on like nursing, with more scope of practice. But that’s not the case.*

## Discussion

The aim of this study was to explore the sources of influence impacting ODP students’ decisions to choose this career. Sources of influence were defined as how different means of learning about the profession had impacted career choice as opposed to how participants were learning about the profession. The study was, to the knowledge of the researchers, the first national questionnaire exploring sources of influence among current ODP students in England. Understanding the different sources of influence which are impacting decisions to choose an ODP career can help with workforce development.

Personal sources of influence were found to be the most impactful, with educational sources the least. Information from universities, previous work in healthcare and role models were all identified as key sources of influence in choosing an ODP career. Gaining an insight into the theatre environment was also identified in the open question as important. Conducting own research was also found to be essential. Researching the profession has also been described as a ‘vital process’ to gain further insights in the study by Ali et al (2022). Although an individual conducting their own research is an understandable source of influence, it is important that other sources of influences can also be impactful.

### Appealing to a younger audience

Only 14% of our sample was under 21. However, Wordsworth (2015) found that 30% of their ODP student sample were under 21 at the time of enrolment on their course, and in 2016/17 over-25s made up 57% of new enrolments to ODP courses in England (Office for Students 2019). These findings may indicate a lack of awareness with students only discovering the profession having left school or college. This suggests a low impact of educational sources which we found in our study and which echoes Wordsworth’s (2015) finding. It is of concern to find that 61.3% of participants in our study reported not to have been influenced by an educational source. Accordingly, more needs to be in place to ensure career advisors and future careers programmes are promoting ODP. Work has been done to increase publicity and understanding of the ODP profession for younger individuals through the WOW Show (2024). In addition, the College of ODPs (n.d.) has a website dedicated to promoting and explaining the ODP career. The Step into the NHS (2023) website and the NHS (n.d.) health careers website both explain the role of an ODP and what ‘everyday life’ for an ODP involves. It would be beneficial to promote these resources to career advisors and future careers programmes to encourage potential new students.

### Exposure to the healthcare setting

It was noticeable that over 50% of our sample viewed a previous job in healthcare as influential (90% for male participants). Furthermore, the open question data confirmed this with a number of participants highlighting their previous employment as theatre support workers or HCAs as a source of influence for learning more about the ODP role. Rodger and Mahoney (2017) explored the transition from HCA to ODP, highlighting the benefits of working in a healthcare setting, and specifically in theatre, allowing for gaining this greater understanding of an ODP and other multidisciplinary team members.

Work experience may relate to more formalised hands-on experience of an area of work, speaking to an individual in the role, or watching an individual perform their job role (which we define as work shadowing for this study). In our study, participants recommended that pre-course experience in or exposure to theatre would help individuals determine whether the ODP role was for them. Ali et al (2022) suggested that unfamiliarity with the theatre environment before a first clinical placement added to ODP course attrition. Work experience in healthcare is ‘preferable’ to apply to a HEI ODP course, although work experience could comprise only speaking to an ODP about the role. In the National Operating Department Practitioner Workforce Programme 2021-22 (HEE 2022), it was highlighted that there is confusion as to whether there is a legal age limit for theatre work experience. Access to work experience for aspiring ODPs varies locally, but there are opportunities for individuals aged under 18 to access theatre work experience. In addition, Springpod (2024) have a virtual work experience (for all allied health professionals) for students living in the South East of England. Furthermore, where work experience is not viable, work shadowing in theatre could act as an alternative and could be promoted more at school/college. In addition, To understand the ODP role, Ali et al (2022) found that students were receptive to simulations and videos to provide a virtual experience of theatres. The use of mock theatres could also be utilised to improve accessibility to the profession (see 350+ NHS Careers, n.d.). In addition, work experience could involve being in theatre after a procedure (as opposed to during the procedure) to gain familiarity of the ODP role and theatre environment.

### ODPs as role models

The importance of personal sources of influence aligns with literature from other AHPs, which has found personal influence to be an important factor in choosing a healthcare career path (Barfield et al 2011, Byrne 2015). This was also echoed in our open question findings: speaking to those who knew the role (qualified ODPs and students) was seen as important before beginning an ODP career. The composition of our sample in terms of gender is also of note with 80.7% of the sample female. Our sample may be an indication of the trajectory of gender balance in the future for the ODP population. The Health and Care Professions Council (2021) found that the ratio at a national level of qualified ODPs was 60% female to 40% male. A large proportion of male participants in our sample had been influenced by an ODP, suggesting that role modelling is especially important for men coming into the profession. It is important to ensure that all ODP professionals are made aware of the important role they play in ensuring future generations of ODPs. The age range in our sample suggests that all interactions with ODPs can be a potential recruitment opportunity.

## Strengths and limitations

Our research allowed for the exploration of ODP students’ views of different sources of influence at a national level, which provides a useful dataset for future studies exploring this topic area.

In terms of limitations, recall ability is a recognised limitation in questionnaires (Althubaiti 2016). Participants may be biased from their current experiences which may have influenced their answers to how they felt when they chose the ODP profession. It should be acknowledged that our sample was self-selecting and therefore cannot be said to represent the ODP student population in England. Although the composition of our sample is similar to findings in other studies (see Wordsworth 2015) and possibly representing the ODP learner population, this comprised only 22 participants under 21 and only 30 male participants. This small sample size means that our findings around gender and age are not generalisable. Finally, it is important to acknowledge that we did not ask participants to disclose whether they were on a degree or diploma course. Capturing this data would have allowed us to explore if there was differences in sources of influences for the two groups; however, from September 2024, the diploma option will no longer be available (see Clayton 2021).

## Conclusion and suggestions for future research

The supply pipeline and retention of ODPs and ODP learners has been recognised as a priority in England (NHS England 2023). Owing to sample size, we were unable to explore in-depth findings for students on the apprenticeship route. But as apprenticeship routes begin to form a larger proportion of ODP students and owing to the disparity in ODP course attrition (HEE 2022), exploring whether there are differences between this group and students on the traditional undergraduate route in terms of sources of influence would be beneficial. Our study found that ODP students are influenced by a number of different sources to choose an ODP career, but the impact of these sources is wide-ranging. Accordingly, there is an opportunity to utilise these sources more effectively to ensure that students are learning about the profession but also gaining an understanding of the role before choosing this profession to help with attrition. The influence of current ODPs as role models, especially for male ODP students, suggests an opportunity to encourage qualified ODPs to become career ambassadors. Further research should explore how educational sources, especially career advisors, could be utilised more to influence those aged under 21 to consider an ODP course. Finally, our study has highlighted the perceived importance of obtaining work shadowing or work experience to prepare for the ODP role. Addressing the misconceptions around age restrictions for theatre work experience while promoting alternative tools, such as virtual work experience, is key.

## Key points

- Exploring the factors which influence individuals to choose an ODP career is an under researched area.
- ODP students are influenced by a number of different sources to choose an ODP career, but the impact of these sources is wide-ranging.
- Personal influences, such as role models, were the key sources of influence for choosing an ODP career.
- The supply pipeline and retention of ODPs and ODP learners has been recognised as a priority in England.
